# Accelerating Burden of Hypertensive Heart Disease in Low- and Lower-Middle-SDI Countries, 1990–2023: The Contribution of Cold Temperature Exposure

**DOI:** 10.5334/aogh.5329

**Published:** 2026-09-11

**Authors:** Mingyang Ruan, Ruixue Zhang, Jinglian Wei, Tiezheng Niu, Shiqi Wang, Wenming Shi, Guangyuan Yang, Tianwei Dong

**Affiliations:** 1 Department of Cardiology, First Affiliated Hospital of Jiamusi University, Jiamusi, 154002, China; 2 Liangping District People’s Hospital, Chongqing, 405200, China; 3 Li Ka Shing Faculty of Medicine, The University of Hong Kong, Hong Kong, 999077, China

**Keywords:** hypertensive heart disease, regional disparities, extreme temperatures, GBD, socio-demographic index, health inequality, BAPC

## Abstract

*Background:* Low- and lower-middle-socio-demographic index (SDI) countries face a rapidly growing burden of hypertensive heart disease (HHD). However, the contribution of non-optimal temperature, particularly cold exposure, remains poorly characterised in these populations.

*Methods:* Using Global Burden of Disease Study 2023 data, we analysed HHD deaths, disability-adjusted life years (DALYs), and age-standardised rates in 79 low- and lower-middle-SDI countries from 1990 to 2023. Temporal trends were assessed using estimated annual percentage change (EAPC), socioeconomic inequality using slope index of inequality (SII) and concentration index, and temperature-attributable burden using the GBD comparative risk assessment framework. Bayesian age–period–cohort models projected burden to 2050.

*Results:* From 1990 to 2023, deaths from HHD in low- and low-middle-SDI countries combined increased by approximately 192% (from 157,950 to 461,110), with corresponding DALYs rising by approximately 171% across these countries. Across all SDI strata, the age-standardizsed mortality rate increased significantly in low-SDI countries (EAPC+ 0.58%; 95% CI: 0.53%–0.63%), whereas the increase in low-middle-SDI countries was not statistically significant (EAPC+ 0.06%; 95% CI: 0.00%–0.12%), with Central Sub-Saharan Africa recording the highest 2023 rate (44.6 per 100,000). Cold temperature consistently contributed a greater temperature-attributable HHD burden than high temperature throughout the study period. Despite the overall increase in HHD burden, the absolute cold-attributable burden increased only modestly, whereas age-standardised cold-attributable rates remained largely stable, particularly in low-SDI settings. Cross-country absolute inequality widened substantially (SII −18.93 to −20.66). Projections indicate that HHD deaths and DALYs will increase markedly by 2050, driven mainly by population ageing among women.

*Conclusion:* Low- and lower-middle-SDI countries face a growing HHD burden, with cold exposure representing an important environmental contributor. Its impact should be considered alongside population ageing, hypertension control, healthcare accessibility, and other cardiovascular determinants. Strengthening hypertension management and implementing low-cost, winter-specific cardiovascular protection strategies may help mitigate projected burden.

## Background

Hypertensive heart disease (HHD) represents a major late-stage consequence of long-standing uncontrolled hypertension and is a leading driver of heart failure and premature mortality worldwide. It ultimately results in heart failure [1, 2]. In low- and lower-middle-SDI countries, HHD is the second leading cause of heart failure, following ischaemic heart disease, accounting for 26.2% of cases [3]. HHD is not only a target organ damage caused by hypertension but also an independent risk factor for the occurrence of stroke, coronary heart disease, and all-cause mortality [2, 4, 5]. In these resource-constrained settings, hypertension awareness, treatment, and control rates remain critically low—often below 30%, 20%, and 10%, respectively—exacerbating HHD burden amid rapid population ageing and limited access to care [6, 7]. In addition to the long-term damage caused by hypertension itself, environmental climate change—particularly fluctuations in extreme cold temperatures—has emerged as a significant contributing factor to the worsening of outcomes in patients with HHD.

Cold exposure elicits sympathetic nervous system activation and peripheral vasoconstriction, which increase systemic vascular resistance and both systolic and diastolic blood pressure. Sustained cold-related pressure elevation raises left ventricular afterload and, together with cold-induced endothelial dysfunction, activation of the renin–angiotensin–aldosterone system, and pro-thrombotic and pro-inflammatory changes, may accelerate left ventricular hypertrophy and adverse cardiac remodelling—the pathological substrate of HHD. In resource-constrained settings, inadequate indoor heating, poor housing insulation, and limited access to anti-hypertensive treatment plausibly amplify these responses [8–10]. Despite overall global declines in age-standardised HHD rates, low- and lower-middle-SDI countries—home to over three billion people—face the opposite trend [11]. This divergence highlights the need to identify context-specific environmental and structural risk factors that may disproportionately affect these regions. However, the frequent occurrence of extreme cold events, such as cold waves, has had a significant impact on the incidence and mortality risk of HHD in these regions, where inadequate heating infrastructure amplifies vulnerability [12].

Despite the fact that epidemiological studies have confirmed the close association between temperature anomalies and HHD [11, 12], critical gaps remain in existing research on extreme temperatures and HHD. First, socioeconomic coverage is inadequate, with a particular scarcity of data in low- and lower-middle-SDI regions, where cold-attributable HHD burden continues to rise while declining elsewhere. Second, most studies over-rely on acute endpoints, with limited exploration of long-term disease burden (e.g. disability-adjusted life years, DALYs) and demographic susceptibility patterns. Third, there is a lack of systematic quantification of health inequalities within these vulnerable strata. These limitations impede evidence-based climate-adaptive HHD prevention. Therefore, this study aims to explore the disease burden of HHD attributable to extreme temperature-related risk factors in low- and lower-middle-SDI countries.

Utilising the 2023 Global Burden of Disease (GBD) database, we systematically analysed the burden of HHD from 1990 to 2023 in 79 low- and lower-middle-SDI countries, with the following primary objectives: (1) to examine differences in HHD-related mortality, DALYs, and age-standardised rates (ASRs) across regional, national, and demographic (age and sex) dimensions, focusing on the impact of extreme temperatures—particularly cold—as a key environmental risk factor; and (2) to quantify temporal trends and health inequalities from 1990 to 2023 using estimated annual percentage change (EAPC), slope index of inequality (SII), and concentration index (CI). In addition, we explored the contributions of demographic and epidemiological drivers and projected future disease burden to inform climate-adaptive HHD prevention strategies in low-resource settings.

## Materials and Methods

### Data source

The latest 2023 GBD database has comprehensively evaluated diseases, injuries, and risk factors across populations of different ages, regions, and genders. The GBD study provides a comprehensive framework for estimating disease burden and risk factors worldwide, including 371 diseases and injuries and 88 risk factors across 204 countries and territories. The methodological framework was based on previously published GBD 2021 studies, while the latest estimates analysed in this study were extracted from the GBD 2023 database, including mortality, DALYs, ASRs, temperature-attributable burden, and socio-demographic index (SDI) values. All estimates underwent standard GBD modelling and uncertainty assessment procedures [9, 13]. Data related to deaths and DALYs for HHD can be viewed and downloaded from the VizHub—GBD Results (IHME) website (https://vizhub.healthdata.org/gbd-results/).

Data for HHD (ICD-10 codes I11–I11.9) were extracted exclusively for the 79 low- and lower-middle-SDI countries using the GBD Results Tool. Selected metrics included: (1) number and ASRs of deaths and DALYs (per 100,000 population) with 95% uncertainty intervals (UIs); (2) low- and high-temperature-attributable deaths and DALYs (Level 4 risk factors in the GBD comparative risk assessment framework); (3) country-level SDI values from 1990 to 2023.

### Definition

Although the GBD 2023 HHD data include all age groups (0–100+ years), this study focused on individuals aged 15 years and older, consistent with the age stratification presented in the results. HHD incidence and mortality are extremely low in children and adolescents, and age-specific estimates in these younger groups are less reliable. Age-standardised rates and decomposition analyses were therefore conducted for the ≥15-year-old population. The selected age range is consistent with the specific age range specified in the GBD 2023 database.

Disability weights (DWs) represent the degree of poor health associated with a specific disease or disability, relative to a healthy state. In the GBD, DWs are primarily used to calculate years lived with disability (YLD) as part of the DALY metric, where the YLD formula is: YLD = disease prevalence × disability weight. This allows YLD to be combined with years of life lost (YLL) due to premature death to form the total DALY [14].

The SDI is a comprehensive index formed by standardising indicators such as education level, fertility rate, and per capita income, and is used to reflect the development level of a country. This study used the most recently available SDI data (reference year: 2023) as the classification standard, and countries were assigned to the corresponding SDI groups (low and low–middle) according to their SDI values for that year. The inclusion criteria were as follows:(1) SDI data availability: countries were required to have available SDI values in the GBD 2023 dataset. (2) Completeness of health burden data: countries had to report HHD deaths and DALYs in GBD 2023 sufficient for calculating ASRs and conducting trend analyses. (3) Acceptable data quality: countries were excluded if data were missing, estimates were unstable, or key indicators lacked sufficiently reliable UIs, to ensure analytical robustness. Based on these criteria and the GBD 2023 SDI values, a total of 79 eligible low-SDI and low-middle-SDI countries and territories were included. These countries were considered to represent the full set of low socio-demographic development settings with complete HHD data available, and were therefore used for cross-national comparisons and temporal analyses. The SDI value ranges from 0 to 1, with a higher value indicating a higher level of development. Based on these criteria, the SDI values of 204 countries and regions are divided into five groups. The latest grouping is as follows: low SDI (<0.45), lower-middle SDI (0.45 ∼ < 0.61), middle SDI (0.61 ∼ < 0.69), upper-middle SDI (0.69 ∼ < 0.80), and high SDI (≥0.80) [15].

The types of HHD studied were extracted using the International Classification of Diseases (ICD-10) codes: HHD(I11–I11.9) [16].

### Statistical analyses

The DALYs and death indicators of the studied HHD were quantified using the ASRs. This method eliminates the impact of differences in age composition, ensuring the comparability of the research indicators [17]. In the GBD database, all data on ASRs and their 95% UI estimates are expressed per 100,000 population. The UI is defined as the 2.5th and 97.5th percentiles obtained from 1000 ordered samplings. The basic calculation method of ASR is as follows:


$$\text{ASR}=\frac{\sum_{i=1}^{A}{a_{i}w}_{i}}{\sum_{i=1}^{A}w_{i}}$$

where $a_{i}$ denotes the age $i^{th}$ age group, $w_{i}$ is the number of people in the standardised population within each age group, *A* represents the number of age groups, and 95% UI is defined as the 2.5th and 97.5th values of the ordered 1000th extraction.

Decomposition analysis was performed to quantify the contributions of population growth, population ageing, and changes in age-specific mortality rates to changes in HHD deaths over time. Demographic decomposition was performed using the Das Gupta method to partition changes in HHD deaths between 1990 and 2023 into three components: population growth, population ageing, and changes in age-specific HHD death rates. Decomposition was conducted for each SDI stratum separately, with results aggregated across countries within each stratum. The interaction term was distributed proportionally between demographic components following standard practice.

Estimates were calculated via a generalised linear regression model to evaluate the EAPCs in ASRs, which relates the natural logarithm (ln) of the ASRs to time, revealing the evolution of the ASRs over time [17, 18]. EAPCs and their 95% CIs were calculated using EAPC = 100 × [exp(*β*) − 1]. The basic calculation of EAPCs is as follows:


Y=α+βX+ℇ

where ln (ASR), *α* denotes the intercept, *X* denotes the calendar year, *ℇ* denotes the error term, and *β* corresponds to the linear positive or negative trend of ASR. EAPC was considered statistically significant if the 95% CI excluded zero.

Socioeconomic inequalities across the 79 countries were quantified using the SII for absolute differences and the CI for relative differences, both based on SDI ranking. A negative SII value indicates that the burden is disproportionately higher in low-SDI countries, whereas a positive value would indicate higher burden in high-SDI countries [19]. Global and high-SDI data are included in Table 1 as reference benchmarks to contextualise the divergent trends observed in low-resource settings.

**Table 1:** Numbers and ASRs of hypertensive heart disease burden and their temporal trends from 1990 to 2023.

LOCATION	1990		2023		1990–2023		1990		2023		1990–2023	
	MORTALITY CASES	ASMR (95% UI)	MORTALITY CASES	ASMR (95% UI)	FOLD CHANGE (1990 = 1)	EAPC (95% CI)	DALYs CASES	ASDR (95% UI)	DALYs CASES	ASDR (95% UI)	FOLD CHANGE (1990 = 1)	EAPC (95% CI)
Global	732.01 (529.24, 893.68)	21.24 (15.61, 25.65)	1487.75 (1192.61, 1809.49)	16.78 (13.48, 20.37)	1.03	−0.67 (−0.84, −0.50)	15654.91 (11288.65, 19292.08)	409.79 (298.17, 504.99)	28504.65 (22891.29, 35295.04)	316.09 (253.86, 391.81)	0.82	−0.79 (−0.96, −0.62)
Sex												
Male	372.36 (275.25, 486.97)	24.76 (18.95, 31.88)	639.31 (507.21, 815.36)	16.42 (13.03, 20.85)	0.72	−1.14 (−1.32, −0.97)	8700.11 (6340.59, 11462.41)	495.68 (368.48, 646.08)	13725.88 (10813.59, 17770.94)	328.43 (260.46, 423.10)	0.58	−1.18 (−1.35, −1.01)
Female	359.65 (225.06, 479.11)	18.32 (11.66, 24.22)	848.44 (644.12, 1118.38)	16.72 (12.68, 22.19)	1.36	−0.28 (−0.44, −0.11)	6954.80 (4301.03, 9300.71)	334.13 (208.78, 445.43)	14778.78 (11033.63, 20127.98)	300.33 (223.07, 410.50)	1.12	−0.39 (−0.57, −0.22)
SDI												
Low SDI	82.25 (53.34, 119.04)	28.72 (18.71, 41.15)	264.11 (175.83, 379.22)	34.62 (22.79, 49.68)	2.21	0.58 (0.53, 0.63)	1977.59 (1285.23, 2938.23)	558.98 (368.83, 812.12)	5970.44 (4043.70, 8428.29)	660.55 (446.53, 934.14)	2.02	0.48 (0.41, 0.55)
Low-middle SDI	75.70 (49.01, 108.22)	25.76 (16.78, 37.44)	197.00 (136.73, 285.02)	25.73 (17.82, 37.47)	1.6	0.06 (0.00, 0.12)	1759.42 (1135.51, 2552.03)	490.37 (319.01, 697.56)	4139.23 (2914.79, 5903.56)	469.71 (330.85, 669.81)	1.35	−0.08 (−0.15, −0.02)
Middle SDI	84.72 (60.08, 110.07)	31.91 (22.69, 41.45)	198.94 (149.15, 258.55)	26.18 (19.67, 34.06)	1.35	−0.64 (−0.75, −0.54)	1973.35 (1397.31, 2565.87)	628.92 (452.62, 810.44)	4392.57 (3334.34, 5628.96)	513.93 (390.61, 662.61)	1.23	−0.63 (−0.72, −0.54)
High-middle SDI	205.74 (126.93, 273.23)	37.87 (23.84, 49.74)	315.53 (231.25, 371.05)	16.93 (12.33, 19.92)	0.53	−2.46 (−2.90, −2.01)	4429.15 (2752.45, 5910.56)	695.00 (436.53, 915.09)	5728.99 (4370.20, 6790.02)	292.26 (221.94, 345.61)	0.29	−2.66 (−3.09, −2.23)
High SDI	279.82 (232.33, 323.17)	14.66 (12.25, 16.92)	502.96 (427.54, 559.88)	10.82 (9.26, 11.99)	0.8	−0.80 (−0.91, −0.69)	5445.24 (4563.93, 6376.52)	269.96 (226.81, 314.64)	8126.78 (7200.36, 8927.40)	186.96 (166.36, 204.96)	0.49	−1.07 (−1.18, −0.97)
Region												
High-income Asia Pacific	17.05 (14.23, 19.50)	10.43 (8.53, 11.98)	25.30 (18.21, 31.28)	3.40 (2.61, 4.16)	0.48	−3.10 (−3.78, −2.41)	281.26 (237.97, 316.93)	156.84 (132.13, 177.52)	315.59 (247.59, 379.10)	52.79 (42.64, 63.90)	0.12	−3.10 (−3.75, −2.45)
High-income North America	23.14 (20.64, 24.97)	6.62 (5.89, 7.14)	85.45 (74.53, 93.40)	12.53 (11.16, 13.63)	2.69	1.99 (1.80, 2.19)	489.29 (447.75, 524.49)	145.73 (133.18, 156.43)	1696.15 (1547.18, 1824.42)	284.21 (260.96, 306.51)	2.47	2.21 (2.06, 2.35)
Western Europe	53.59 (46.77, 59.50)	8.91 (7.74, 9.93)	109.95 (86.54, 124.13)	8.36 (6.73, 9.38)	1.05	0.30 (0.10, 0.49)	817.38 (732.76, 893.12)	136.87 (122.75, 149.85)	1342.71 (1128.69, 1486.17)	113.57 (97.72, 124.66)	0.64	−0.11 (−0.27, 0.05)
Australasia	0.75 (0.66, 0.81)	3.48 (3.03, 3.80)	1.57 (1.31, 1.76)	2.27 (1.93, 2.53)	1.1	−1.10 (−1.45, −0.74)	12.74 (11.54, 13.80)	56.70 (50.83, 61.56)	23.69 (20.66, 26.01)	38.61 (34.20, 42.03)	0.86	−1.01 (−1.41, −0.61)
Andean Latin America	2.34 (1.94, 2.83)	13.23 (10.97, 16.14)	5.28 (4.33, 6.67)	8.70 (7.12, 10.98)	1.26	−1.22 (−1.52, −0.91)	46.67 (38.71, 55.65)	234.11 (194.55, 279.04)	95.21 (80.92, 117.11)	150.74 (127.97, 184.85)	1.04	−1.20 (−1.52, −0.89)
Tropical Latin America	16.95 (15.11, 18.90)	22.20 (19.50, 24.90)	30.44 (26.63, 33.21)	11.64 (10.15, 12.71)	0.8	−1.76 (−1.88, −1.64)	390.19 (354.77, 430.19)	439.13 (396.38, 485.73)	582.90 (525.75, 624.91)	218.84 (196.92, 234.56)	0.49	−1.98 (−2.10, −1.87)
Central Latin America	12.69 (11.75, 13.53)	18.11 (16.66, 19.38)	25.10 (22.50, 27.82)	10.20 (9.12, 11.32)	0.98	−1.86 (−2.10, −1.62)	247.31 (231.17, 262.07)	318.43 (297.97, 338.21)	432.88 (393.21, 472.83)	168.26 (152.77, 183.96)	0.75	−2.03 (−2.31, −1.75)
Southern Latin America	7.34 (6.56, 8.17)	18.29 (16.22, 20.40)	15.31 (13.30, 17.06)	14.50 (12.68, 16.11)	1.09	−0.66 (−0.82, −0.51)	132.79 (120.54, 146.68)	305.07 (275.25, 337.73)	218.26 (195.30, 241.09)	218.01 (196.34, 240.52)	0.64	−0.95 (−1.10, −0.81)
Caribbean	4.74 (4.17, 5.40)	18.33 (16.20, 20.84)	13.80 (12.08, 16.02)	24.40 (21.31, 28.34)	1.91	1.20 (0.93, 1.46)	102.14 (89.30, 118.30)	381.63 (334.00, 442.07)	281.27 (242.89, 332.74)	502.51 (432.67, 595.72)	1.75	1.11 (0.85, 1.37)
Central Europe	31.34 (29.45, 33.24)	23.63 (21.98, 25.14)	62.30 (56.02, 66.76)	26.45 (23.77, 28.37)	0.99	0.69 (0.50, 0.87)	589.74 (557.70, 623.86)	412.78 (389.42, 437.04)	973.97 (897.89, 1037.87)	425.77 (394.08, 452.60)	0.65	0.41 (0.23, 0.58)
Eastern Europe	13.03 (11.37, 14.58)	5.01 (4.37, 5.62)	25.07 (22.54, 27.58)	7.03 (6.30, 7.75)	0.92	1.21 (0.45, 1.98)	313.18 (274.47, 349.78)	113.92 (99.60, 127.32)	460.71 (419.36, 505.33)	128.42 (116.83, 141.18)	0.47	0.24 (−0.54, 1.02)
Central Asia	6.82 (5.90, 7.88)	17.82 (15.47, 20.64)	13.05 (11.41, 15.09)	18.42 (16.10, 21.29)	0.91	0.56 (−0.05, 1.18)	148.01 (128.87, 169.12)	342.01 (296.89, 391.96)	266.00 (231.62, 305.67)	326.47 (284.84, 375.40)	0.8	0.01 (−0.63, 0.65)
North Africa and Middle East	60.54 (39.89, 84.20)	48.97 (32.21, 67.44)	150.50 (118.19, 196.64)	40.82 (31.91, 53.50)	1.49	−0.57 (−0.62, −0.52)	1366.35 (895.52, 1890.87)	898.87 (597.58, 1233.62)	3067.58 (2383.50, 3942.34)	700.75 (551.33, 907.62)	1.25	−0.78 (−0.83, −0.72)
South Asia	90.04 (51.10, 137.97)	21.86 (12.59, 33.70)	317.62 (205.27, 460.24)	26.04 (16.77, 38.06)	2.53	0.72 (0.65, 0.80)	2096.65 (1194.12, 3161.86)	407.65 (236.30, 620.58)	6488.07 (4259.78, 9355.43)	457.08 (301.50, 657.07)	2.09	0.49 (0.41, 0.57)
Southeast Asia	52.61 (32.91, 77.03)	24.48 (15.29, 35.67)	127.70 (84.51, 174.38)	19.59 (12.92, 26.58)	1.43	−0.73 (−0.78, −0.68)	1283.34 (799.48, 1910.61)	514.39 (323.67, 755.65)	3085.33 (2081.71, 4191.37)	430.28 (291.06, 585.59)	1.4	−0.54 (−0.59, −0.49)
East Asia	282.36 (167.11, 399.37)	44.82 (26.64, 62.61)	316.87 (209.31, 391.06)	14.41 (9.42, 17.89)	0.12	−3.62 (−4.24, −3.00)	5935.03 (3531.80, 8587.70)	780.49 (472.19, 1099.86)	5252.19 (3690.38, 6301.93)	230.14 (159.85, 275.57)	−0.12	−3.91 (−4.48, −3.34)
Oceania	0.67 (0.46, 0.96)	28.55 (19.79, 39.71)	1.67 (1.13, 2.42)	24.43 (16.68, 35.89)	1.5	−0.73 (−0.79, −0.66)	19.04 (13.03, 27.16)	632.75 (437.91, 899.23)	47.46 (32.36, 70.29)	549.66 (375.31, 793.47)	1.49	−0.65 (−0.72, −0.58)
Western Sub-Saharan Africa	16.96 (10.71, 24.93)	23.04 (14.86, 33.89)	44.50 (27.86, 65.01)	23.34 (14.68, 34.12)	1.62	−0.17 (−0.24, −0.09)	438.58 (273.85, 637.63)	495.23 (315.04, 721.04)	1213.60 (765.34, 1754.13)	522.10 (332.78, 748.30)	1.77	−0.00 (−0.10, 0.09)
Eastern Sub-Saharan Africa	23.82 (13.72, 36.93)	37.96 (22.33, 58.80)	74.09 (45.37, 110.53)	41.31 (25.43, 60.98)	2.11	0.17 (0.10, 0.24)	572.04 (323.89, 903.99)	735.59 (431.84, 1130.17)	1659.15 (1029.66, 2475.57)	810.85 (503.39, 1201.05)	1.9	0.22 (0.15, 0.30)
Central Sub-Saharan Africa	6.42 (3.61, 10.54)	39.44 (23.17, 64.64)	21.84 (12.22, 34.34)	44.60 (24.89, 70.65)	2.4	0.22 (0.11, 0.33)	175.82 (97.78, 282.62)	794.66 (458.61, 1297.22)	559.04 (322.10, 857.84)	900.36 (512.99, 1406.13)	2.18	0.24 (0.11, 0.37)
Southern Sub-Saharan Africa	8.81 (6.61, 11.50)	36.27 (27.28, 47.06)	20.33 (15.63, 26.14)	33.89 (25.94, 43.93)	1.31	−0.24 (−0.46, −0.01)	197.37 (147.74, 252.94)	729.40 (548.81, 941.83)	442.91 (343.81, 557.87)	671.99 (517.69, 848.48)	1.24	−0.28 (−0.52, −0.04)

To quantify the contribution of ambient temperature to HHD burden, we extracted estimates from the GBD 2023 Comparative Risk Assessment framework. In the GBD framework, temperature-attributable burden represents population-level burden estimated from modelled temperature exposure distributions, exposure–response relationships, and the theoretical minimum risk exposure level (TMREL). The population attributable fraction is calculated based on these parameters and subsequently applied to total HHD deaths and DALYs to estimate the burden associated with non-optimal temperature exposure. Therefore, temperature-attributable estimates should be interpreted as risk attribution at the population level rather than evidence that temperature exposure independently determines temporal changes in HHD burden. Detailed descriptions of temperature exposure assessment, TMREL estimation, lag structures, and exposure–response modelling (including distributed lag non-linear models) have been reported in previous GBD publications and methodological documentation. In this study, we did not independently perform temperature exposure modelling or risk estimation; instead, all attributable burden estimates were obtained directly from the GBD database. Uncertainty intervals (95% UIs) were derived from 1000 posterior draws provided by the GBD study.

To project future burden while avoiding implausible extrapolation of ASRs under rapid demographic change, we used Bayesian age–period–cohort (BAPC) models with default settings to estimate multiplicative effects of age, period, and cohort. BAPC models were used to project future age-standardised HHD rates by sex, incorporating age, period, and cohort effects. Absolute numbers of deaths and DALYs were subsequently derived by applying projected age-specific rates to World Health Organization (WHO) medium variant population forecasts. Age, period, and cohort effects were modelled using second-order random walk priors (RW2) with non-informative hyperpriors on precision. Model convergence was assessed using Potential Scale Reduction Factor (PSRF < 1.1) and Deviance Information Criterion (DIC). Back-testing was performed by fitting the model to data up to 2018 and predicting 2019–2021, which confirmed that the model adequately captured observed trends. Posterior distributions were summarised to obtain median estimates and 50%, 80%, and 95% credible intervals. Although specific numerical point estimates for ASRs are not reported in tables, the posterior median and UIs are visualised in Figure 7 to illustrate projected trends. Models were fitted to observed 1990–2023 data and extrapolated using the WHO medium variant population forecasts for the 79 countries [20, 21]. Projections were limited to absolute numbers of deaths and DALYs. No specific point estimates for ASRs by sex in 2050 were reported. All analyses and visualisations were performed in R version 4.4.2 using the BAPC and INLA packages.

## Results

### HHD burden at the global, regional, and national levels in 2023

**Figure 1:**
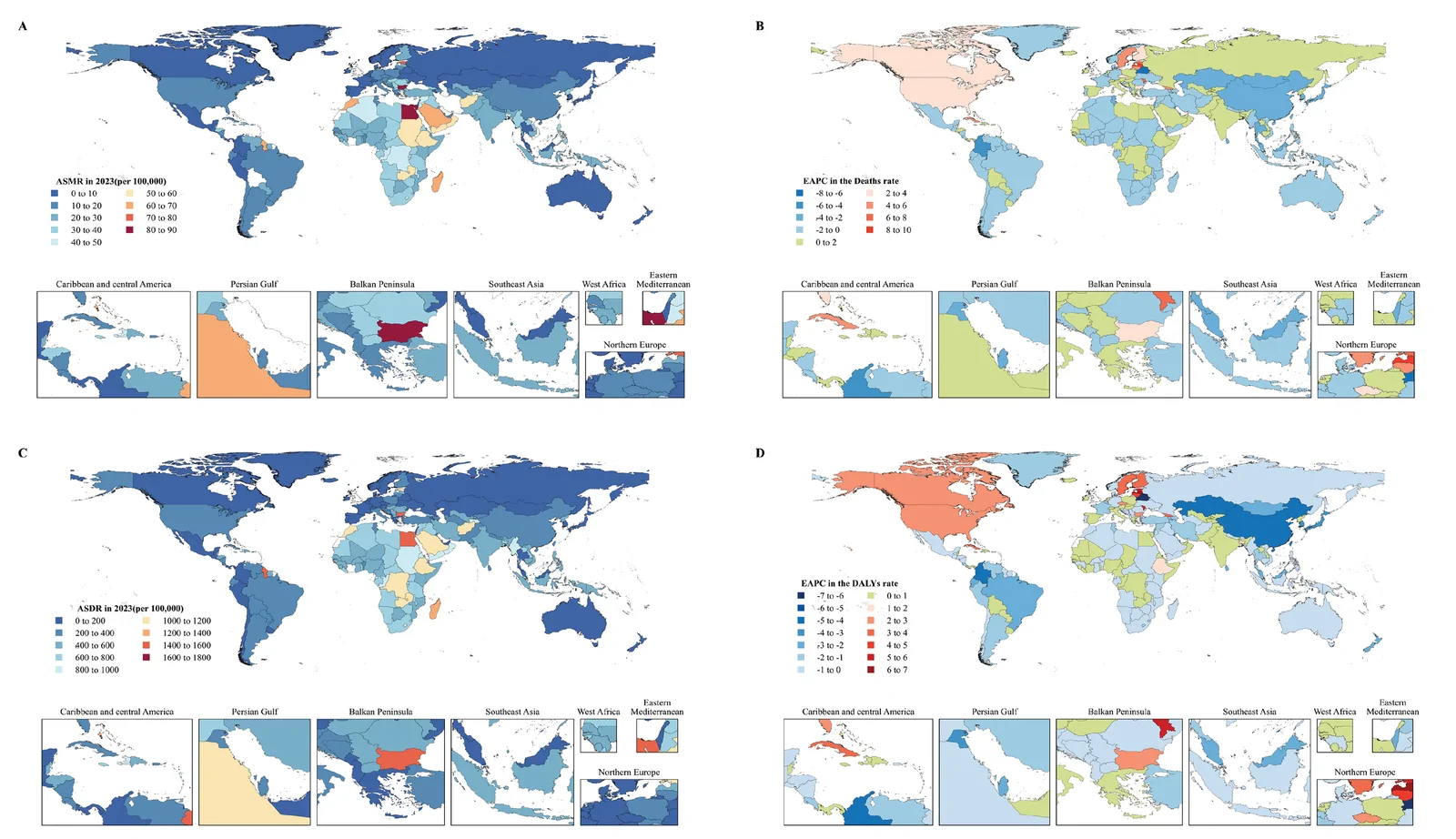
Global disease burden of HHD in 204 countries or territories in 2023. **(A, C)**: Age-standardised mortality rate (ASMR, per 100,000) and DALY rate (ASDR, per 100,000), with darker colours indicating higher rates. **(B, D)**: Estimated annual percentage change (EAPC) in ASMR and ASDR; negative values indicate decline, positive values indicate increase. Insets show regional variations in selected areas.

In 2023, substantial geographical heterogeneity was observed in the ASRs of HHD, with the highest burdens concentrated in Central Sub-Saharan Africa and other low-SDI regions (Figure 1).

**Figure 2:**
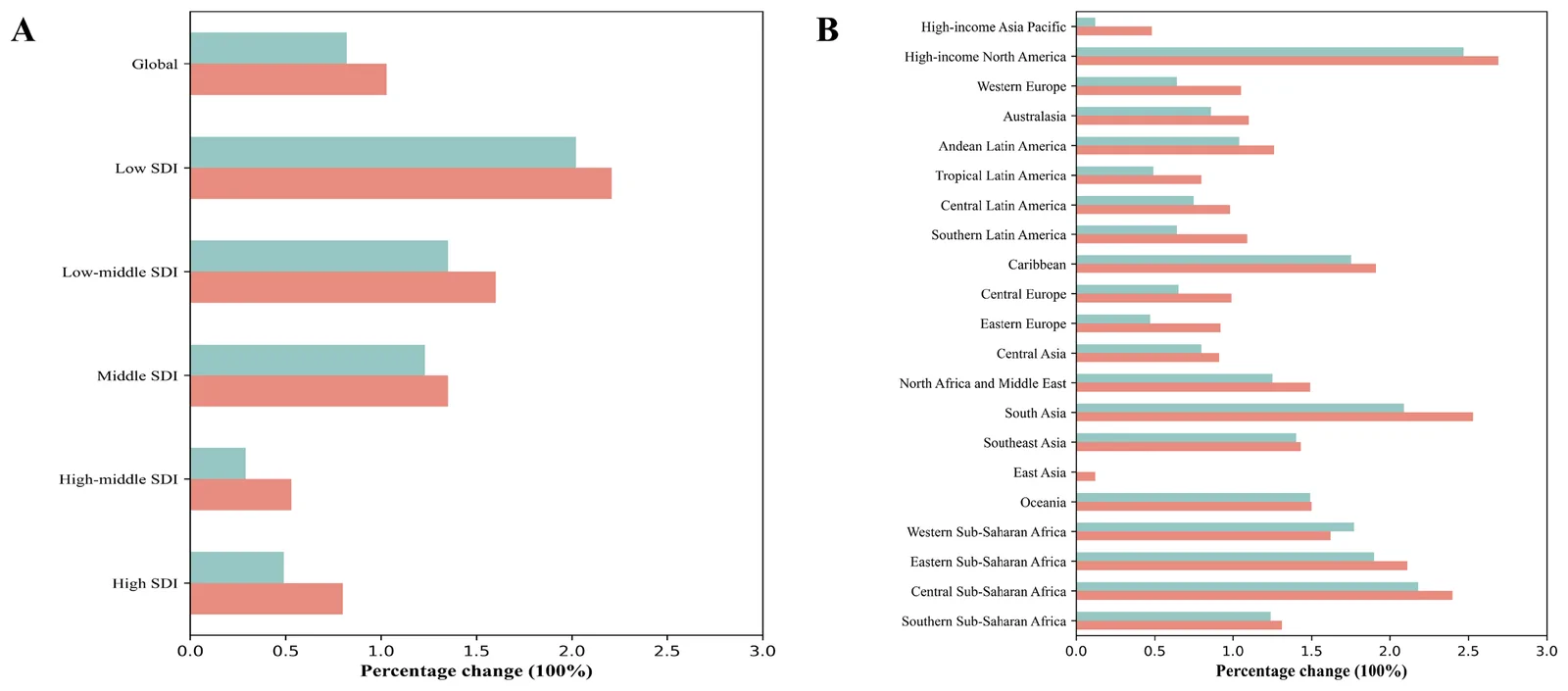
Percentage changes in HHD burden by SDI category and global region, 2023 compared with 1990. **(A)** Percentage changes in HHD burden across Socio-demographic Index (SDI) categories. **(B)** Percentage changes in HHD burden across 21 global regions. Bars indicate the percentage change in HHD burden from 1990 to 2023. Red bars represent changes in the number of deaths, and green bars represent changes in DALYs.

Compared with 1990, the low-SDI region showed the most significant growth in the number of deaths and DALYs in 2023 (221%). The number of cases and ASRs of HHD deaths and DALYs in 1990 and 2023 are detailed in Table 1 and Figure 2.

**Figure 3:**
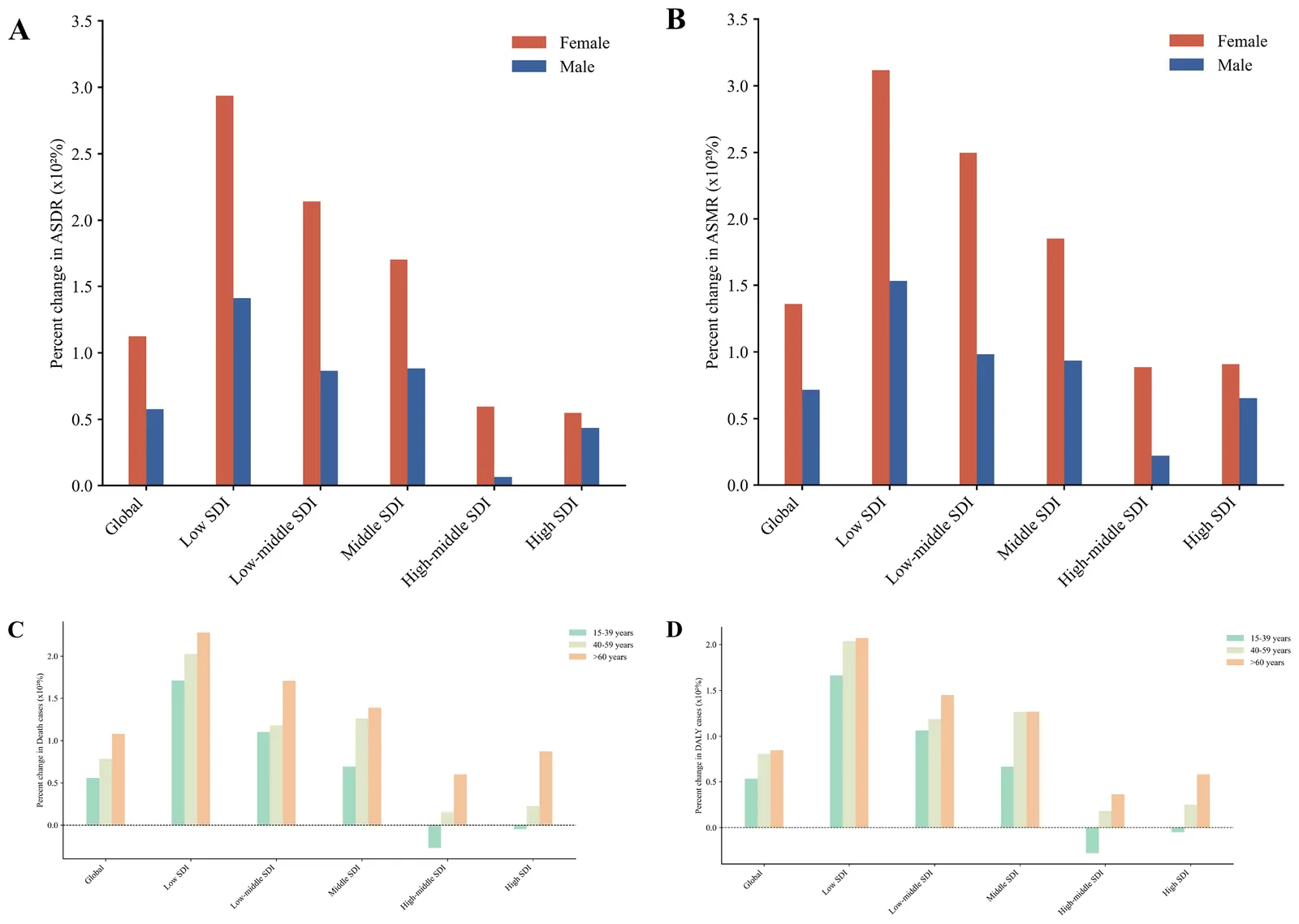
Percentage changes in HHD by sex and age group across SDI regions. **(A)** ASDR by sex. **(B)** ASMR by sex. **(C)** ASDR by age group. **(D)** ASMR by age group. SDI, sociodemographic index; ASDR, age-standardised DALY rate; DALYs, disability-adjusted life years.

By 2023, however, the region with the highest ASR shifted to Central Sub-Saharan Africa, with an age-standardised mortality rate (ASMR) of 44.60 (24.89, 70.65) and an age-standardised DALY rate (ASDR) of 900.36 (512.99, 1406.13) (Table 1; Figure 2). The burden increased progressively with age across all SDI regions, reaching the highest levels in those aged 95 years and older. Among SDI regions, males and females in the low-SDI region had the highest disease burden in terms of crude deaths and DALYs (Table 1; Figure 3), reflecting the combined effects of high prevalence of untreated hypertension and advanced population ageing in these settings.

### Trends in HHD burden from 1990 to 2023

**Figure 4:**
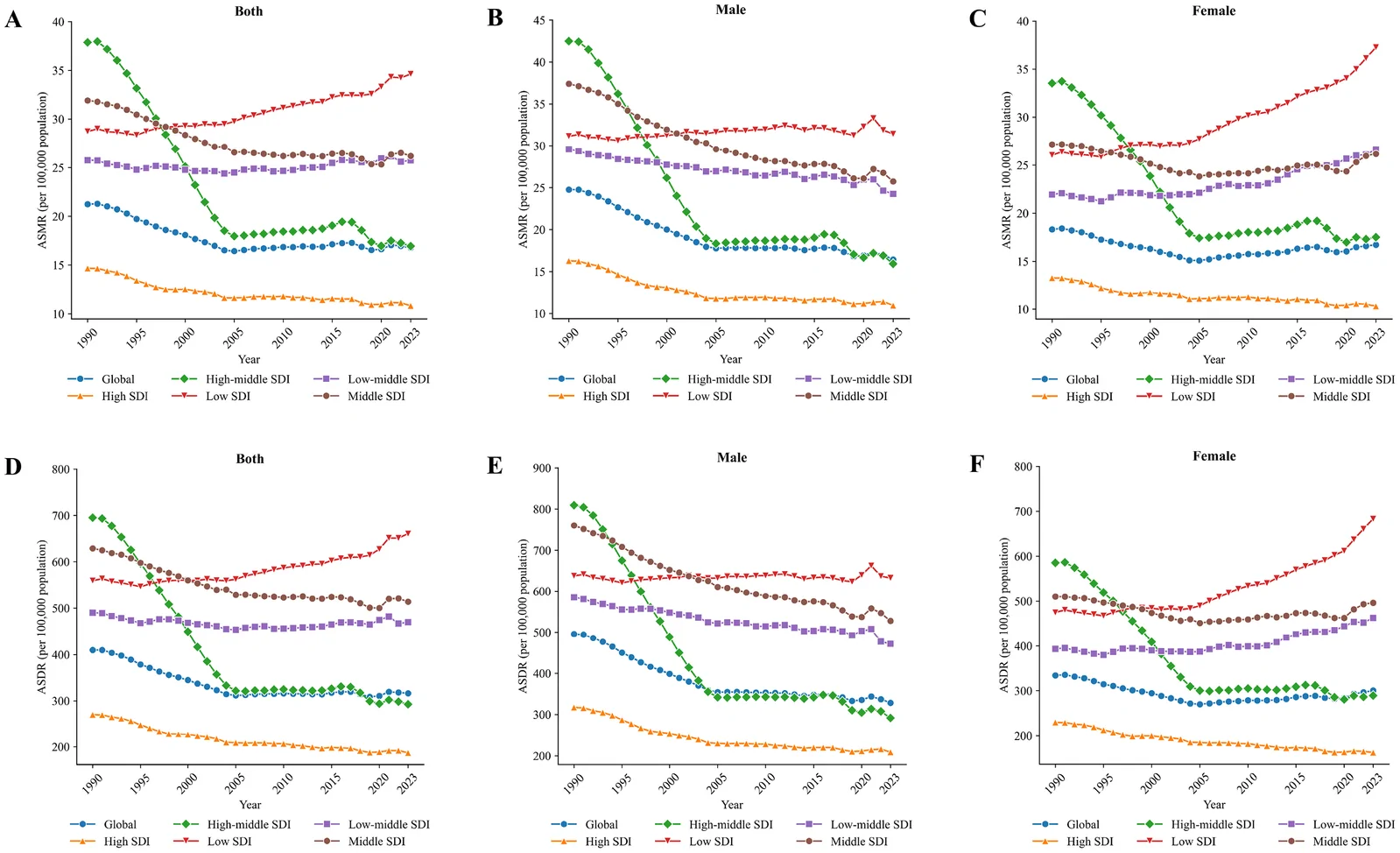
Trends in global HHD burden changes from 1990 to 2023. **(A, B, C)** Age-standardised mortality rate (ASMR, per 100,000) for both sexes, males, and females, respectively, by SDI category. **(D, E, F)** Age-standardised DALY rate (ASDR, per 100,000) for both sexes, males, and females, respectively, by SDI category. Lines represent different SDI levels as indicated in the legend.

Globally, the absolute burden of HHD rose substantially from 1990 to 2023 due to population growth and ageing, but ASRs generally declined, reflecting improvements in hypertension management in many settings (Table 1; Figure 4).

EAPCs of global ASMR and ASDR for HHD calculated from 1990 to 2023 were −0.67% (95% CI: −0.84, −0.50) and −0.79% (95% CI: −0.96, −0.62), respectively, both showing a downward trend. In contrast, both indicators were positive in low-SDI countries, whereas in lower-middle-SDI countries only the ASMR showed a slight increase. The combined number of absolute deaths in low- and lower-middle-SDI countries increased by approximately 192% (from 157,950 to 461,110), with DALYs rising by approximately 171% (Table 1; Figure 4).

These divergent trends highlight the persistent challenge of hypertension control in low-resource settings and underscore the growing contribution of low- and lower-middle-SDI countries to the global HHD burden.

### Decomposition analysis of HHD burden

From 1990 to 2023, ageing and population growth led to an increase in the death burden, while epidemiological changes resulted in a decrease in the death burden. In the gender-specific analysis, the decomposition results for males were consistent with the overall global trend (for both genders). However, for females, epidemiological changes, ageing, and population growth all contributed to the increase in the death burden.

**Figure 5:**
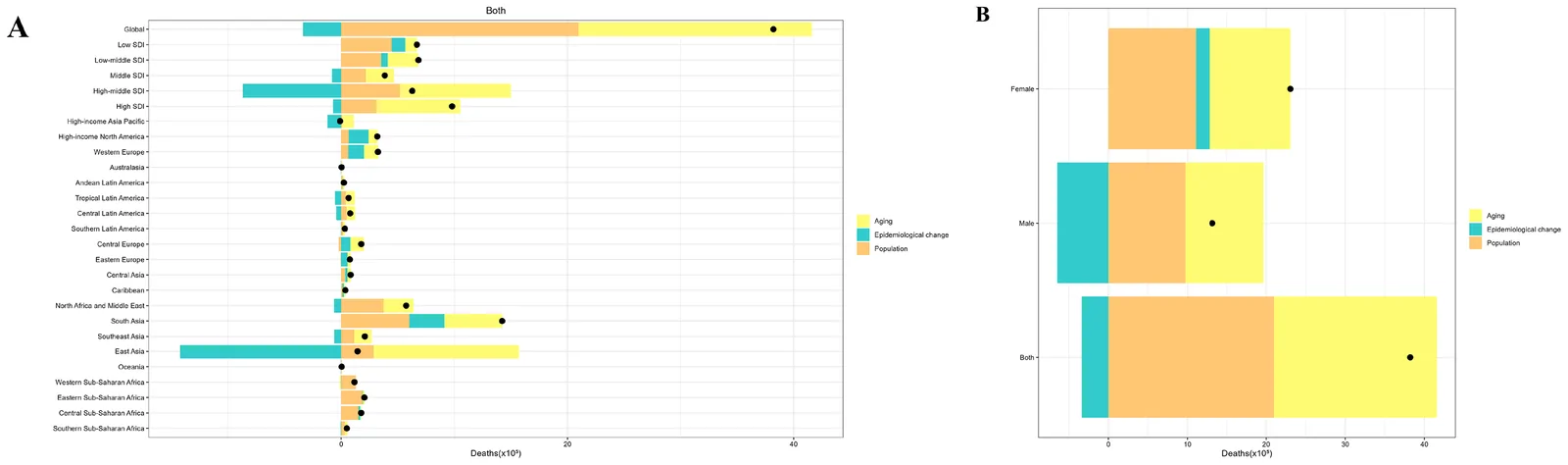
Regional and gender-specific decomposition analyses of HHD burden. **(A)** 21 GBD regions (both sexes combined). **(B)** By sex. Bars represent contributions from ageing (yellow), epidemiological change (teal), and population growth (orange); black dots indicate total change.

Contrary to the global trend, age-specific mortality rates in low-SDI countries exhibited an upward trajectory over the study period, thereby contributing positively to the overall increase in HHD-related deaths. Decomposition analysis within these regions indicated that population ageing accounted for approximately 35% of the rise, population growth contributed roughly 25%, and alterations in age-specific HHD mortality rates explained nearly 40% of the total increase in deaths between 1990 and 2023. In low-middle SDI regions, the corresponding contributions were approximately 30% from ageing, 20% from population growth, and 50% from changes in age-specific mortality rates. These estimates, derived from decomposition analysis, reflect the relative contributions of demographic and epidemiological factors to changes in HHD mortality (Figure 5). Notably, East Asia, which lies outside the primary focus on low- and lower-middle SDI regions, showed a disproportionately large absolute contribution (∼40 × 10 deaths) in the global decomposition figure. This is largely attributable to its large population size and rapid demographic ageing rather than higher per-capita HHD risk. Therefore, this regional contribution should be interpreted cautiously and not as a focal finding of the present study.

Across all SDI regions, epidemiological changes generally reduced burden in line with the global pattern. Notably, in low- and lower-middle-SDI regions, epidemiological improvements had a weaker mitigating effect, resulting in positive contributions from all drivers in some strata and underscoring the limited impact of preventive advances in resource-constrained environments (Figure 5).

### Health inequality analysis of HHD burden

In 1990 and 2023, the analysis of absolute inequality in HHD across countries worldwide showed that the SII (from −18.93 to −20.66) for HHD in 2023 was more negative than that in 1990. This indicates that, over this period, the inequality in ASMR burden, driven by differences in SDI levels, increased. As a result, a higher HHD burden is now more prevalent in countries with lower SDI rankings.

**Figure 6:**
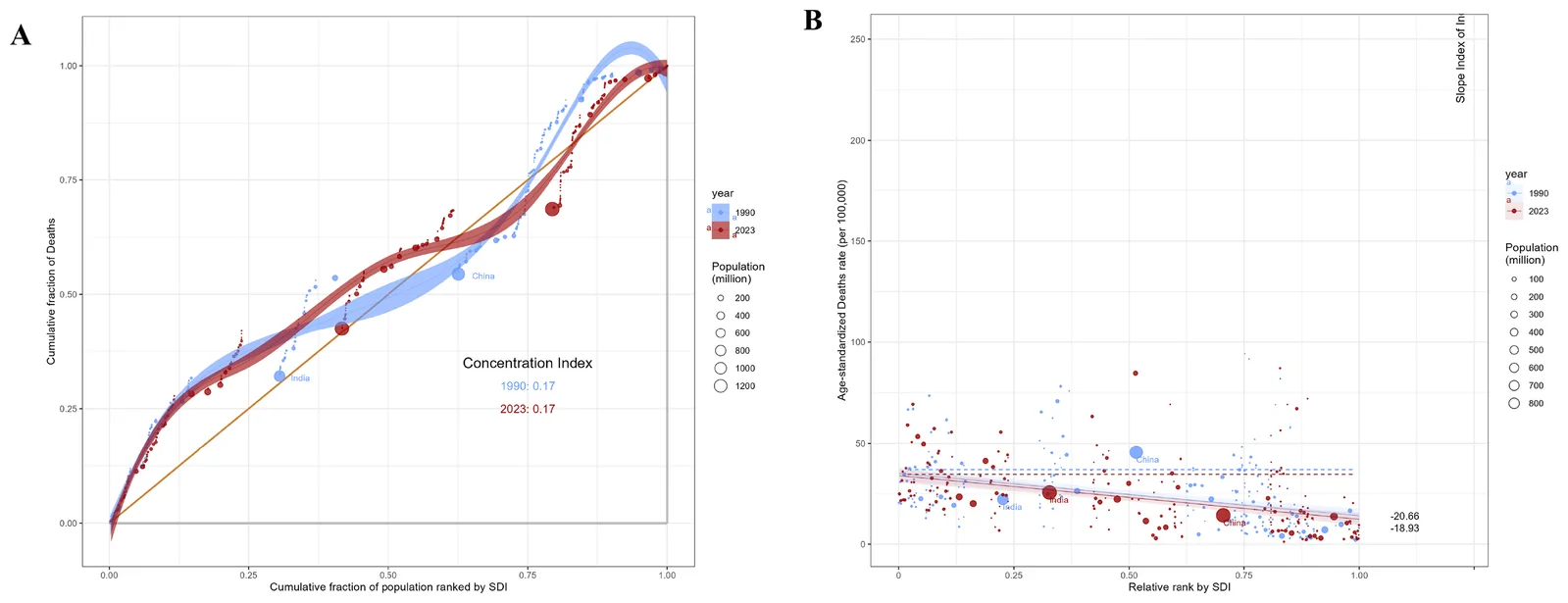
Health inequality analysis of HHD-related deaths (1990 and 2023). **(A)** Lorenz curves showing cumulative HHD deaths by SDI-ranked population; concentration index values are indicated. **(B)** Relationship between age-standardised death rate and SDI rank; bubble size represents population, and trend lines show slope indices of inequality.

Relative inequality, measured by the CI, remained stable over the period, indicating no reduction in the proportional disadvantage faced by lower-SDI countries (Figure 6).

### Projected HHD burden from 2024 to 2050

**Figure 7:**
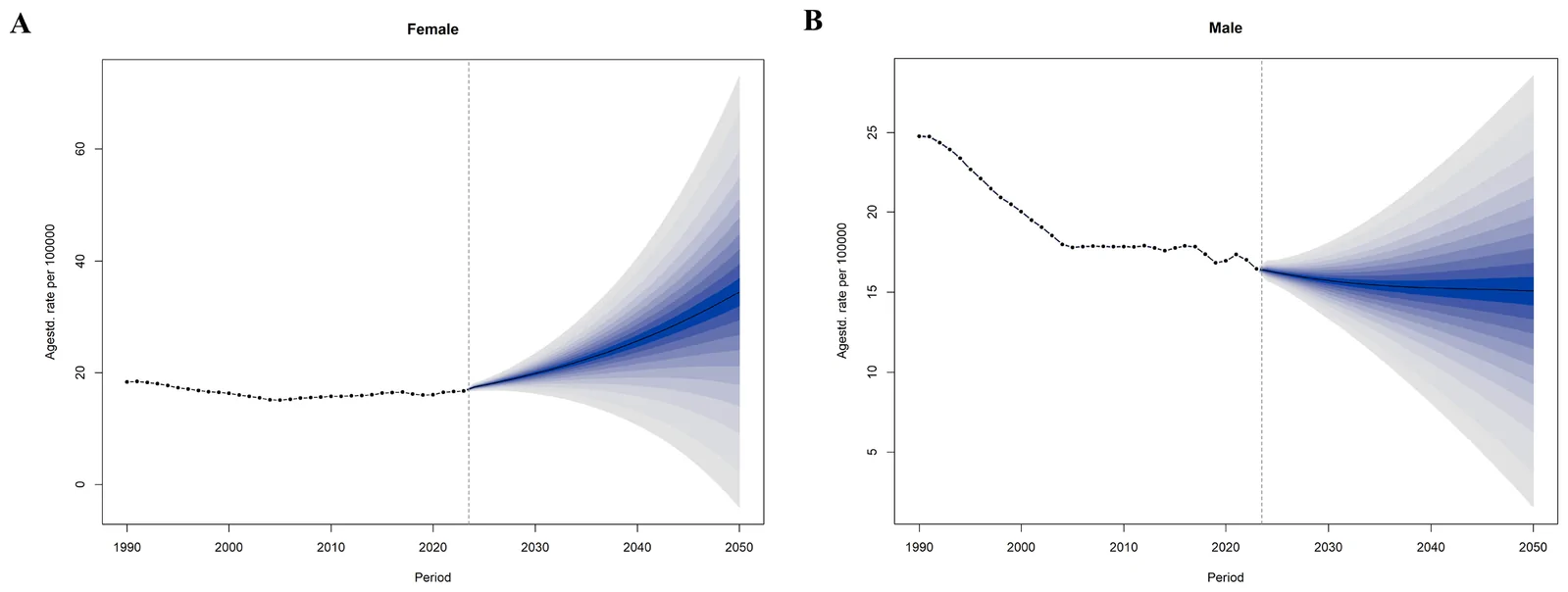
Projections of HHD burden from 2024 to 2050. **(A, B)** BAPC model projections of age-standardised HHD rates per 100,000 population among females and males, respectively, from 2024 to 2050. Solid lines indicate posterior median estimates, and shaded areas represent posterior credible intervals (50%, 80%, and 95%).

The BAPC projections indicate that absolute numbers of HHD deaths and DALYs in low- and lower-middle-SDI countries will increase substantially by 2050 under continuation of current trends (Figure 7). This rise is driven predominantly by population ageing and a growing proportion of elderly women, who face higher vulnerability due to longer life expectancy, lower historical rates of hypertension screening, and reduced postmenopausal cardiovascular protection. These patterns highlight the urgent need for targeted, gender-responsive prevention strategies in resource-constrained settings to avert a widening burden of preventable heart failure.

### HHD burden attributable to non-optimal temperature

**Figure 8:**
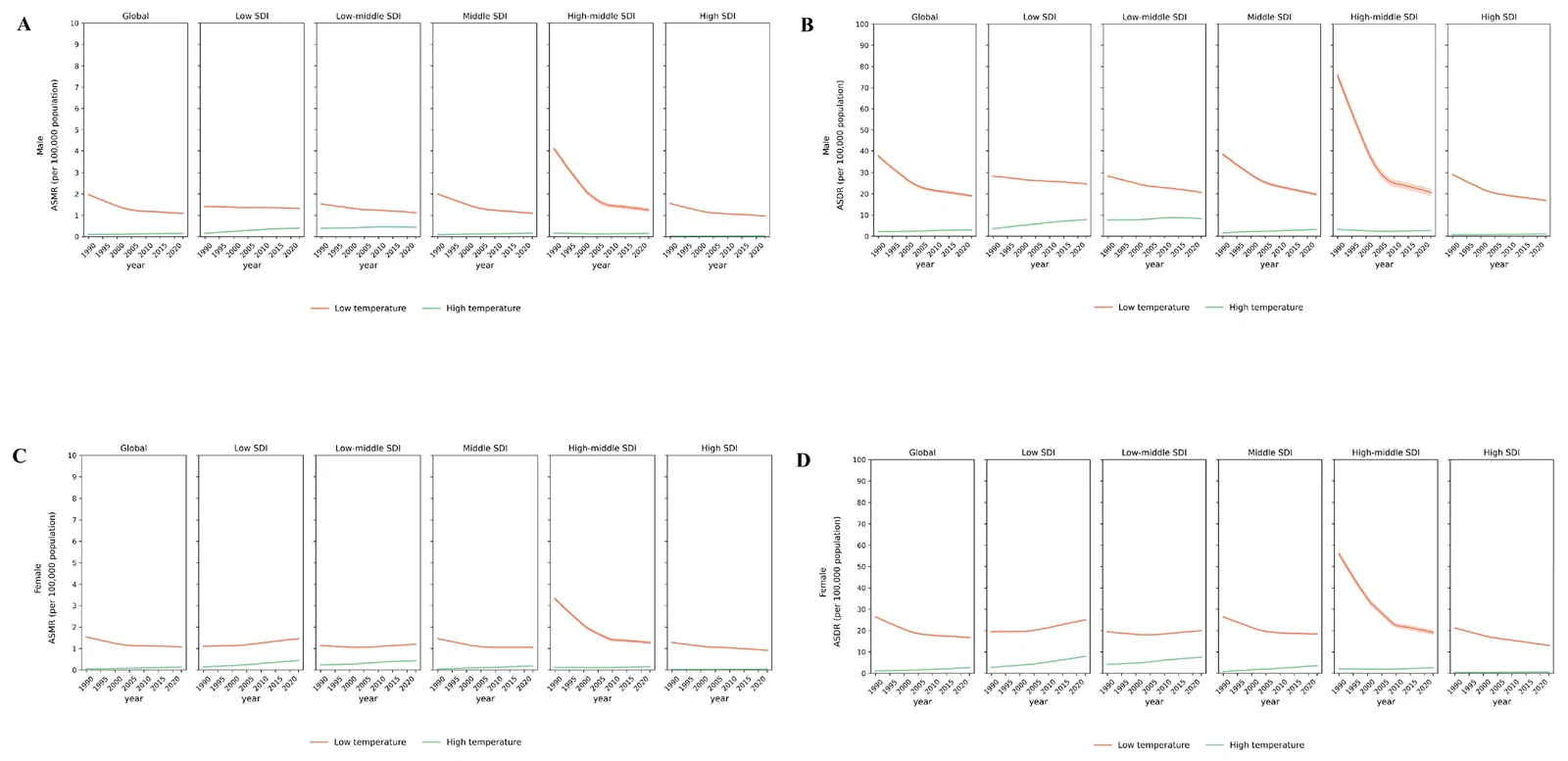
Trends in HHD burden changes influenced by low temperature and high temperature from 1990 to 2023. **(A, C)** Age-standardised mortality rate (ASMR) for males and females, respectively, by SDI category, stratified by low and high temperature. **(B, D)** Age-standardised DALY rate (ASDR) for males and females, respectively, by SDI category, stratified by low and high temperature. Lines indicate temperature-specific contributions to HHD burden.

To explore the changes in the impact of environmental factors on HHD burden across different regions, we analysed the trends in risk factors related to HHD from 1990 to 2023. In this study, in low-SDI and lower-middle-SDI regions, cold temperature consistently accounted for a greater temperature-attributable HHD burden than high temperature throughout the study period, indicating a substantial contribution of cold exposure to temperature-related cardiovascular burden, with only minor fluctuations between 1990 and 2023. In contrast, the burden attributable to hot temperature gradually increased over the same period, contributing to the overall upward trend. In high-middle SDI regions, cold temperature-attributable HHD burden exhibited a sharp decline around the early 2000s, with ASMRs dropping from ∼4 per 100,000 to ∼1–2 per 100,000, before stabilising thereafter. This abrupt decline likely reflects improvements in hypertension management and healthcare access, as well as potential artefacts from sparser historical data. In high SDI regions, both cold- and hot temperature-attributable burdens remained relatively stable throughout the study period (Figure 8).

## Discussion

In low- and lower-middle-SDI countries, the burden of HHD has shown sharply increasing absolute numbers. Low SDI was the only SDI stratum in which both ASMR and ASDR increased significantly, whereas lower-middle SDI showed only a marginal increase in ASMR (EAPC 0.06%, 95% CI: 0.00–0.12) and a slight decline in ASDR [11, 14]. From 1990 to 2023, deaths increased by 192% and DALYs by 171%, underscoring the challenges posed by insufficient hypertension control, limited access to blood pressure monitoring, and inadequate availability of anti-hypertensive medications. Notably, within this group, low-SDI regions, particularly Central Sub-Saharan Africa, experienced the largest increase in deaths (221%), while DALYs increased by 202%, the highest among all SDI strata, reflecting severe constraints on healthcare infrastructure and the high prevalence of untreated hypertension [17]. These findings highlight the need for region-specific public health strategies that prioritise strengthening hypertension control and improving cardiovascular disease management to reduce the HHD burden and related health inequalities.

Beyond the long-term damage caused by hypertension, exposure to extreme cold represents an important environmental contributor to the HHD burden. Cold temperatures induce vasoconstriction and elevate blood pressure, thereby increasing cardiac workload, effects that may be amplified by inadequate heating infrastructure and limited physiological adaptive capacity in resource-poor settings. Cold-attributable HHD ASRs have remained persistently high in low- and lower-middle-SDI regions rather than declining with overall improvements in healthcare, further exacerbating health inequalities, whereas higher-SDI regions have benefited from improved housing conditions and climate resilience measures [22]. Although global mean temperatures are rising, increased climate variability may sustain episodic cold exposure in low-SDI regions, potentially explaining why cold-attributable age-standardised HHD rates have remained persistently high rather than declining. However, this hypothesis requires further investigation using high-resolution climate and health data [9, 23]. These findings underscore the importance of integrating climate-sensitive cardiovascular prevention strategies, including community-based winter warming initiatives and seasonal reinforcement of anti-hypertensive treatment.

Our projections indicate a striking sex difference in the future burden of HHD. While male rates remained relatively stable, female rates showed a modest projected increase. Despite the relatively stable ASMRs, the absolute number of HHD deaths and DALYs in low- and lower-middle-SDI countries is projected to increase markedly by 2050, due primarily to population growth and ageing. These projections, based on BAPC modelling using GBD 2023 data, indicate that elderly women in low- and lower-middle-SDI countries are likely to experience a disproportionate increase in HHD burden compared to men. This sex difference is driven by biological factors, including loss of cardioprotective oestrogen after menopause, as well as social and behavioural factors, such as lower awareness, treatment, and management of hypertension, and differences in comorbidity profiles and healthcare access. These findings underscore the need for targeted, gender-sensitive interventions, including blood pressure screening, early diagnosis, anti-hypertensive treatment, and community programmes addressing modifiable risk factors. Incorporating sex-specific projections into HHD prevention policies and resource allocation is essential to mitigate the growing burden among women in these settings.

From 1990 to 2023, the SII for age-standardised HHD rates increased in magnitude, indicating a widening absolute disparity in disease burden across low- and lower-middle-SDI countries. Persistent inequities in access to hypertension diagnosis, treatment, and long-term follow-up continue to drive these disparities, particularly in rural and marginalised communities where diagnostic capacity and medication availability remain limited [24]. This intra-group inequality highlights that socioeconomic gradients remain a critical determinant of cardiovascular outcomes even within the world’s poorest countries.

Future projections indicate substantial increases in the absolute HHD burden, driven largely by population ageing and a growing proportion of elderly women, who often experience lower lifetime rates of hypertension screening, delayed treatment initiation, and reduced cardiovascular protection after menopause. These patterns highlight the need for sex- and age-specific prevention strategies, including gender-responsive screening programmes and improved continuity of hypertension care in primary healthcare settings.

Several limitations should be acknowledged. First, GBD estimates rely on country-level data, and data completeness may be limited in some low-SDI countries, affecting the precision of regional and inequality analyses. Second, associations between extreme temperature exposure and HHD were assessed using observational modelling, which may be influenced by residual confounding from factors like air pollution and dietary patterns. Future prospective studies and intervention trials are needed to clarify causal pathways. Additionally, SII and CI were applied at the country level using national SDI values, which may not fully reflect within-country socioeconomic disparities and should be interpreted as cross-national rather than intra-population inequality indices. Furthermore, the temperature-attributable burden in the GBD framework is based on short-term exposure–response relationships and should not be seen as evidence for a causal role of temperature in chronic HHD development. Finally, the 33-year span and potential policy changes may result in non-linear trends in HHD rates, and a single EAPC may not capture important inflection points, particularly in regions with non-monotonic trends (e.g. high-middle SDI).

Although cold temperature accounted for a greater temperature-attributable burden than high temperature, these findings do not indicate that cold exposure is the primary driver of increasing HHD burden. The rising burden observed in low- and lower-middle-SDI countries is likely attributable to multiple interacting factors, including population ageing, population growth, inadequate hypertension awareness and treatment, limited healthcare accessibility, socioeconomic disadvantage, and environmental exposures. Therefore, cold temperature should be considered an important and potentially modifiable environmental contributor within a broader framework of cardiovascular risk prevention.

## Conclusion

In summary, HHD remains characterised by substantial and widening health inequalities, particularly in low- and lower-middle-SDI countries. Addressing these disparities requires integrated, multifactorial, climate-adaptive, and equity-oriented public health strategies that prioritise vulnerable populations, including older adults, socioeconomically disadvantaged groups, and individuals with poorly controlled hypertension. Targeted interventions, including affordable fixed-dose combination therapy, community-based hypertension screening, and improved long-term hypertension management, are essential for reducing future HHD burden and inequalities. In addition, climate-adaptive cardiovascular strategies, including winter-specific risk management among vulnerable populations, may provide complementary benefits in settings where cold temperature exposure contributes substantially to HHD burden [25].

## Figures and Tables

**Table:** 

HHD	Hypertensive Heart Disease
GBD	Global Burden of Disease
DALYs	Disability-Adjusted Life Years
ASRs	Age-Standardised Rates
ASMR	Age-Standardised Mortality Rate
ASDR	Age-Standardised DALY Rate
SDI	Socio-Demographic Index
BAPC	Bayesian Age–Period–Cohort
EAPC	Estimated Annual Percentage Change
SII	Slope Index of Inequality
CI	Concentration Index
DWs	Disability Weights
YLD	Years Lived with Disability
YLL	Years of Life Lost
ICD-10	International Classification of Diseases, 10th Revision
UI	Uncertainty Interval
IHME	Institute for Health Metrics and Evaluation
WHO	World Health Organization

## Data Availability

The datasets analysed during the current study are publicly available from GBD 2023 database at https://vizhub.healthdata.org/gbd-results/.

## References

[r1] Drazner MH. The progression of hypertensive heart disease. Circulation. 2011;123:327–334. doi:10.1161/circulationaha.108.845792. 21263005

[r2] Khalique OK, Bello NA. Are we getting closer to the HEART of hypertensive heart disease? Hypertension (Dallas, Tex.: 1979). 2019;74:257–259. doi:10.1161/hypertensionaha.119.13169. 31256720 PMC6938583

[r3] Bragazzi NL, Zhong W, Shu J, et al. Burden of heart failure and underlying causes in 195 countries and territories from 1990 to 2017. Eur J Prev Cardiol. 2021;28:1682–1690. doi:10.1093/eurjpc/zwaa147. 33571994

[r4] Vakili BA, Okin PM, Devereux RB. Prognostic implications of left ventricular hypertrophy. Am Heart J. 2001;141:334–341. doi:10.1067/mhj.2001.113218. 11231428

[r5] Ruilope LM, Schmieder RE. Left ventricular hypertrophy and clinical outcomes in hypertensive patients. Am J Hypertens. 2008;21:500–508. doi:10.1038/ajh.2008.16. 18437140

[r6] Schutte AE, Srinivasapura Venkateshmurthy N, Mohan S, Prabhakaran D. Hypertension in low- and middle-income countries. Circ Res. 2021;128:808–826. doi:10.1161/circresaha.120.318729. 33793340 PMC8091106

[r7] Geldsetzer P, Manne-Goehler J, Marcus ME, et al. The state of hypertension care in 44 low-income and middle-income countries: A cross-sectional study of nationally representative individual-level data from 1·1 million adults. Lancet (London, England). 2019;394:652–662. doi:10.1016/s0140-6736(19)30955-9. 31327566

[r8] Alahmad B, Khraishah H, Royé D, et al. Associations between extreme temperatures and cardiovascular cause-specific mortality: Results from 27 countries. Circulation. 2023;147(1):35–46. doi:10.1161/CIRCULATIONAHA.122.061832. 36503273 PMC9794133

[r9] Gasparrini A, Guo Y, Hashizume M, et al. Mortality risk attributable to high and low ambient temperature: A multicountry observational study. Lancet. 2015;386(9991):369–375. doi:10.1016/S0140-6736(14)62114-0. 26003380 PMC4521077

[r10] Li Y, Wu J, Xu Y, et al. Cold exposure and the cardiovascular system: From physiological adaptation to pathological risk. Front Physiol. 2026;16:1740919. doi:10.3389/fphys.2025.1740919. PMID: ; PMCID: . 41574187 PMC12819719

[r11] Lu M, Li D, Hu Y, et al. Persistence of severe global inequalities in the burden of hypertension heart disease from 1990 to 2019: Findings from the global burden of disease study 2019. BMC Public Health. 2024;24:110. doi:10.1186/s12889-023-17573-9. 38184560 PMC10771693

[r12] Xu C, Nie X, Xu R, Han G, Wang D. Burden trends and future predictions for hypertensive heart disease attributable to non-optimal temperatures in the older adults amidst climate change, 1990-2021. Front Public Health. 2024;12:1525357. doi:10.3389/fpubh.2024.1525357. 39830174 PMC11738906

[r13] GBD 2019 Injuries Collaborators. Global, regional, and national burden of injuries, and burden attributable to injuries risk factors, 1990 to 2019: Results from the Global Burden of Disease study 2019. Public Health. 2024;237:212–231. doi:10.1016/j.puhe.2024.06.011. 39454232

[r14] GBD 2021 Diseases and Injuries Collaborators. Global incidence, prevalence, years lived with disability (YLDs), disability-adjusted life-years (DALYs), and healthy life expectancy (HALE) for 371 diseases and injuries in 204 countries and territories and 811 subnational locations, 1990–2021: A systematic analysis for the Global Burden of Disease Study 2021. Lancet. 2024;403:2133–2161. doi:10.1016/S0140-6736(24)00757-8. 38642570 PMC11122111

[r15] Global Burden of Disease Collaborative Network. Global Burden of Disease Study 2023 (GBD 2023) Socio-Demographic Index (SDI) 1950–2023. Institute for Health Metrics and Evaluation (IHME); 2025. https://ghdx.healthdata.org/record/gbd-2023-socio-demographic-index-sdi.

[r16] GBD 2019 Stroke Collaborators. Global, regional, and national burden of stroke and its risk factors, 1990–2019: A systematic analysis for the Global Burden of Disease Study 2019. Lancet Neurol. 2021;20:795–820. doi:10.1016/S1474-4422(21)00252-0. 34487721 PMC8443449

[r17] Brauer M, Roth GA, Aravkin AY, et al. Global burden and strength of evidence for 88 risk factors in 204 countries and 811 subnational locations, 1990–2021: A systematic analysis for the Global Burden of Disease Study 2021. Lancet. 2024;403:2162–2203. doi:10.1016/S0140-6736(24)00933-4. 38762324 PMC11120204

[r18] Clegg LX, Hankey BF, Tiwari R, Feuer EJ, Edwards BK. Estimating average annual per cent change in trend analysis. Stat Med. 2009;28:3670–3682. doi:10.1002/sim.3733. 19856324 PMC2843083

[r19] Wagstaff A, Paci P, van Doorslaer E. On the measurement of inequalities in health. Soc Sci Med. 1991;33(5):545–557. doi:10.1016/0277-9536(91)90212-U. 1962226

[r20] Knoll M, Furkel J, Debus J, Abdollahi A, Karch A, Stock C. An R package for an integrated evaluation of statistical approaches to cancer incidence projection. BMC Med Res Methodol. 2020;20:257. doi:10.1186/s12874-020-01133-5. 33059585 PMC7559591

[r21] GBD 2021 Forecasting Collaborators. Burden of disease scenarios for 204 countries and territories, 2022-2050: A forecasting analysis for the Global Burden of Disease Study 2021. Lancet (London, England). 2024;403:2204–2256. doi:10.1016/s0140-6736(24)00685-8. 38762325 PMC11121021

[r22] Ma C, Yang J, Nakayama SF, et al. Cold spells and cause-specific mortality in 47 japanese prefectures: A systematic evaluation. Environ Health Perspect. 2021;129:067001. doi:10.1289/ehp7109. 34128690 PMC8204943

[r23] Zhu H, Wang Y, Duan J et al. Increasing hourly population exposure to moderate cold under climate warming. Int J Disaster Risk Sci. 2026;17:253–268. doi:10.1007/s13753-026-00702-4.

[r24] NCD Risk Factor Collaboration (NCD-RisC). Worldwide trends in hypertension prevalence and progress in treatment and control from 1990 to 2019: A pooled analysis of 1201 population-representative studies with 104 million participants. Lancet (London, England). 2021;398:957–980. doi:10.1016/s0140-6736(21)01330-1. 34450083 PMC8446938

[r25] Agarwal A. Fixed-dose combination therapy for the prevention of atherosclerotic cardiovascular disease. Nat Med. 2024;30:1199–1209. doi:10.1038/s41591-024-02896-w. 38532223 PMC11031293

